# A New Decade of Veterinary Research: Societal Relevance, Global Collaboration, and Translational Medicine

**DOI:** 10.3389/fvets.2015.00001

**Published:** 2015-02-26

**Authors:** Mary M. Christopher

**Affiliations:** ^1^Department of Pathology, Microbiology, and Immunology, School of Veterinary Medicine, University of California–Davis, Davis, CA, USA

**Keywords:** one health, animal research, environment, public health, zoonosis, comparative medicine

Veterinary research and clinical contributions reach into every aspect of biomedical health and science: livestock production and food safety; zoonotic diseases, epidemiology, and public health; comparative basic and translational research; companion animal medicine and surgery; animal welfare and the human-animal bond; and wildlife and ecosystem health. Diversity is not only a key strength of veterinary science but also a challenge. Veterinary research in the next decade must strengthen the scientific impact of its core mission in animal health while firmly reinforcing its societal and global relevance. Key research challenges include water- and food-borne pathogens and drug residues, zoonotic pathogens and infectious diseases, and evidence-based companion animal medicine and its translational applications to human health. Meeting these challenges will require cross-disciplinary global collaborations, significant national research investment, and innovative online publishing tools to facilitate networking and open scientific exchange.

## A Glance Over Our Shoulder: Veterinary Research 2010–2014

Future research builds on past achievements and responds to emerging needs. Veterinary research publications in the past 5 years provide a snapshot of recent accomplishments and serve as a pivot point for identifying future challenges. A Web of Science search (2010–2014) was done to identify top-cited articles, group authors, and funding agencies in the veterinary science research area (1). The search was conducted on all databases to identify veterinary research within a broad context, and then on the Web of Science Core Collection for a more specific veterinary focus. Citations are only one metric of scientific impact, but were used here to identify research areas receiving relatively high attention from other researchers; they were calculated per year so recent articles were not disadvantaged.

Highly cited articles fell into three major areas that point to challenges for veterinary research in the future: environmental threats to animal and human health, pathogens and zoonoses, and comparative medicine and pathology (Table 1). These areas stress the global reach of veterinary science and the strong momentum of the One Health Initiative, which emphasizes interconnectedness between animal, human, and environmental health (2). They also reflect a growing emphasis on diseases of companion animals and their expanding role as translational models of human disease.

**Table 1:** **Top-cited veterinary science articles in the Web of Science (2010–2014)**.

Title	Journal (year)	Cites/year
**(A) ALL DATABASES, VETERINARY SCIENCES**		
Emerging fungal threats to animal, plant and ecosystem health	*Nature* (2012)	82.7
Novel orthobunyavirus in cattle, Europe, 2011	*Emerg Inf Dis* (2012)	73.7
*Escherichia coli* O25b-ST131: a pandemic, multiresistant, community-associated strain	*J Antimicrob Chemother* (2011)	55.3
Middle east respiratory syndrome coronavirus in dromedary camels: an outbreak investigation	*Lancet Inf Dis* (2014)	55.0
Infectivity, transmission, and pathology of human-isolated H7N9 influenza virus in ferrets and pigs	*Science* (2013)	49.5
Present and future arboviral threats	*Antiviral Res* (2010)	46.6
Leptospira and leptospirosis	*Vet Microbiol* (2010)	45.8
Methicillin-resistant *Staphylococcus aureus* with a novel mecA homologue in human and bovine populations in the UK and Denmark: a descriptive study	*Lancet Inf Dis* (2011)	45.8
Human betacoronavirus 2c EMC/2012-related viruses in bats, Ghana and Europe	*Emerg Inf Dis* (2013)	39.0
An emerging disease causes regional population collapse of a common North American bat species	*Science* (2010)	38.4
Islet amyloid polypeptide, islet amyloid, and diabetes mellitus	*Physiol Rev* (2011)	36.0
**(B) CORE COLLECTION, VETERINARY SCIENCES**		
Epizootic of ovine congenital malformations associated with Schmallenberg virus infection	*Tijdschr Diergeneeskde* (2012)	22.7
Organ distribution of Schmallenberg virus RNA in malformed newborns	*Vet Microbiol* (2012)	20.0
Reference value advisor: a new freeware set of macroinstructions to calculate reference intervals with microsoft excel	*Vet Clin Pathol* (2011)	13.0
Emergence of porcine epidemic diarrhea virus in the United States: clinical signs, lesions, and viral genomic sequences	*J Vet Diag Invest* (2013)	13.0
From “one medicine” to “one health” and systemic approaches to health and well-being	*Prev Vet Med* (2011)	12.8
Brucellosis at the animal/ecosystem/human interface at the beginning of the 21^st^ century	*Prev Vet Med* (2011)	11.8
Methicillin-resistant *Staphylococcus aureus* in horses and horse personnel: an investigation of several outbreaks	*Vet Microbiol* (2010)	10.6
Detection and molecular analysis of West Nile virus infections in birds of prey in the eastern part of Austria in 2008 and 2009	*Vet Microbiol* (2011)	10.3
ASVCP reference interval guidelines: determination of *de novo* reference intervals in veterinary species and other related topics	*Vet Clin Pathol* (2012)	10.3
Classification of canine malignant lymphomas according to the World Health Organization criteria	*Vet Pathol* (2011)	10.0
**(C) CORE COLLECTION, VETERINARY NON-INFECTIOUS**		
Interventions for atopic dermatitis in dogs: a systematic review of randomized controlled trials	*Vet Dermatol* (2010)	9.8
Prevalence and risk factors for canine epilepsy of unknown origin in the UK	*Vet Record* (2013)	9.0
Markers of stemness in equine mesenchymal stem cells: a plea for uniformity	*Theriogenol* (2011)	8.8
Nutrition, immune function and health of dairy cattle	*Animal* (2013)	8.5
Evaluation of the effects of dietary supplementation with fish oil omega-3 fatty acids on weight bearing in dogs with osteoarthritis	*J Am Vet Med Assoc* (2010)	7.8
Mortality in North American dogs from 1984 to 2004: an investigation into age-, size-, and breed-related causes of death	*J Vet Int Med* (2011)	7.8
A morphologic study of 608 cases of canine malignant lymphoma in France with a focus on comparative similarities between canine and human lymphoma morphology	*Vet Pathol* (2010)	7.6
An epidemiological study of environmental factors associated with canine obesity	*J Sm Anim Pract* (2010)	7.2
Veterinary surgeons and suicide: a structured review of possible influences on increased risk	*Vet Record* (2010)	6.8
Safety and efficacy of a xenogeneic DNA vaccine encoding for human tyrosinase as adjunctive treatment for oral malignant melanoma in dogs following surgical excision of the primary tumor	*Am J Vet Res* (2011)	6.8

Funding was led by Brazilian government ministries and Sao Paolo Research Foundation, which together supported 66 veterinary publications, compared with the European Union (29), UK Department for Environment, Food, and Rural Affairs (24), and US National Institutes of Health (18). Several articles funded by the Korean government and National Research Foundation and the National Natural Science Foundation of China suggest that increased veterinary research output from Asia is poised to continue (3).

## Common Ground: Environmental Threats

Animals and humans share the same environment. Threats to environmental quality by pharmaceutical and pesticide contamination of food and water, and evolving bacterial drug resistance, including methicillin-resistant *Staphylococcus aureus* (MRSA), jeopardize human and animal health and will continue to present a major research challenge for veterinary scientists in the next decade (4). Potential health risks for people who work closely with animals are a top priority. Research also is needed to identify sources of animal and veterinary drugs; assess the role of animals as reservoirs of resistant pathogens; establish interspecies transmission routes (including human to animal); determine risk factors and epidemiology; monitor and adapt agricultural practices; standardize laboratory testing of food pathogens and drug residues; and develop new and effective treatments. Aquaculture-related environmental residues and impact are also a priority (5). Environmental threats require a global collaborative approach by veterinarians, physicians, microbiologists, pharmacologists, toxicologists, analytical chemists, and environmental scientists, as well as a focus on rapidly developing and highly populated countries such as China and India to address agricultural practices, environmental degradation, and their links with animal and human health (6).

## Tracking Pathogens: Zoonotic and Infectious Diseases

Animals and humans share pathogens. One need only consider the origin of the Ebola epidemic: fruit bats living in a hollow tree in Guinea (7). Zoonotic and infectious diseases – bacterial, viral, fungal, protozoal, and parasitic – will continue to dominate veterinary research efforts on a global basis, including novel research on parasite communities (8), small animal infectious diseases, and emerging zoonotic threats posed by rotaviruses, hepatitis E virus, and coronaviruses (Table 1). Animal infections such as white-nose disease of bats, Schmallenberg virus in livestock, and microsporidia in honeybees (9) underlie the very sustainability of agriculture and of wildlife populations. The role of climate change and other anthropogenic factors on emerging pathogens, such as chytridiomycosis in amphibians (10) is a key research priority.

Infectious and zoonotic diseases intersect with the realities of porous borders, regional conflicts, and a range of agricultural, economic, and political systems that affect veterinary and public health. Outbreaks of foot-and-mouth disease and mutations in avian influenza virus in Egypt exemplify the difficulty many countries face in disease surveillance and vaccine compliance. Consortia such as Partners for Rabies Prevention, Cysticercosis Working Group of Peru, and Emerging Babesioses and One Health are important strategies for galvanizing global research support, expertise, and resources. The $100 million PREDICT project brings together ecosystem, wildlife, and disease experts is an unprecedented effort to detect and respond to viruses that move among people, livestock, and wildlife (11).

## Companion Animal Medicine and Pathology: The New Translational Model

Animals and humans share disease concerns, from diabetes to cancer to kidney disease. Continued specialization of companion animal practice – dogs, cats, and horses – has emerged as a parallel microcosm of its human counterpart. Research that advances high quality, evidence-based veterinary practice is essential for improving clinical outcomes and quality of life. Challenges include coordination of clinical trials and multi-institutional studies, improved database quality, and implementation of reporting guidelines (12). Technological advances in diagnostic imaging, laboratory testing, surgical techniques, epidemiologic modeling, and drug therapy of companion animals are accelerating and globalizing. The past 5 years have seen a surge in evidence-based practice guidelines ranging from dental care for dogs and cats (13) to quality assurance in clinical laboratories (Table 1) to advanced life support and CPR (14). These guidelines promote evidence-based medicine, raise the level of veterinary care, and stimulate discussion within the profession.

Companion animal medicine and pathology is the new frontier of translational medicine, engaging both veterinary and medical scientists in the real-world clinical laboratory of naturally occurring disease in animals. While veterinary researchers have long relied on medical research as a basis for investigation and therapy in animals, medical researchers now increasingly look to veterinary research and veterinarians to gain insights and develop translational models of human disease. Important recent examples include novel therapies for melanoma and lymphoma; obesity, nutritional and metabolic disease; aging and degenerative diseases such as osteoarthritis; behavioral and cognitive disorders; and regenerative medicine and stem cell therapy (Table 1). The Veterinary Comparative Oncology Working Group (15) and the comparative sequencing program of the National Institutes of Health (16) exemplify the productive collaborations possible between veterinary and medical clinicians and scientists.

## An Emerging Frontier: Veterinary and Social Science Research

Animals and humans share a social context. The interface between animals and humans in mental health, cancer detection, war, elder care, legal guardianship, and domestic abuse highlights the diverse societal relevance of veterinary science outside traditional biomedical fields and warrants scientifically rigorous research. In addition to well-established research on the human–animal bond, veterinary research also must contribute to the scientific discussion on human euthanasia, healthcare economics, and disaster management and planning. More research is also needed on people who live and work with animals to address addiction, stress, and suicide risk (Table 1), moral behavior, and the cultural, religious, and ethical issues affecting animal and human health and welfare.

## Publishing Tools to Advance Veterinary Research

*Frontiers in Veterinary Science* is a networked, collaborative, open access journal dedicated to the communication, discussion, and dissemination of all aspects of veterinary research. The journal’s core mission in animal health embraces the One Health concept in all of its specialty sections, from Animal Behavior and Welfare to Zoological and Aquatic Medicine. *Frontiers in Veterinary Science* is closely linked with medicine, public health, and environmental and social science domains, facilitating a seamless, integrative approach to biomedical research that emphasizes the societal relevance and global reach of veterinary science while highlighting new developments in veterinary practice and their translational potential for human medicine.

Open access and online publishing open exciting new ways to share, discuss, and publish veterinary research. The Frontiers collaborative peer review system engages authors and reviewers cooperatively and enhances editors’ ability to bring cross-disciplinary expertise to manuscript evaluation. Post-publication commenting enables “audience participation” and social networking redefines research communities. These novel approaches help us think about and solve today’s interconnected biomedical problems in new ways.

Challenges in the next decade of veterinary research, including environmental threats, pathogens, and comparative medicine, will be driven by societal relevance, global engagement, and the need for new translational models. With the help of online publishing innovations such as *Frontiers in Veterinary Science*, the unique contributions made by veterinary science will be both reinforced and integrated within the broader context of biomedical and social sciences, united in the shared goal of improving the health of animals, people, and the planet.

## Conflict of Interest Statement

The author declares that the research was conducted in the absence of any commercial or financial relationships that could be construed as a potential conflict of interest.
